# COVID-19 cases, hospitalizations and deaths after vaccination: a cohort event monitoring study, Islamic Republic of Iran

**DOI:** 10.2471/BLT.22.288073

**Published:** 2022-06-22

**Authors:** Ali Hosseinzadeh, Sajad Sahab-Negah, Sairan Nili, Roqayeh Aliyari, Shahrbanoo Goli, Mohammad Fereidouni, Ali Alami, Mohsen Shati, Elham Ahmadnezhad, Shiva Mehravaran, Mansooreh Fateh, Hamidreza Khajeha, Zahra Emamian, Elahe Behmanesh, Sepideh Mahdavi, Mostafa Enayatrad, Parvin Mangolian shahrbabaki, Alireza Ansari-Moghaddam, Abtin Heidarzadeh, Fariba Shahraki-Sanavi, Seyed Mohammad Hashemi Shahri, Mahlagha Dehghan, Mohammadreza Amini Moridani, Hossein Sheibani, Maryam Abbaszadeh, Reza Jafari, Maryam Valikhani, Ehsan Binesh, Hamid Vahedi, Reza Chaman, Rozita Khodashahi, Mahnaz Amini, Farahzad Jabbari Azad, Fariborz Rezaeitalab, Saeid Amel Jamehdar, Ali Eshraghi, Hamid Sharifi, Seyed Mehdi Hashemi Bajgani, Amin Mahdavi, Abdollah Jafarzadeh, Mehrdad Farokhnia, Saeedeh Ebrahimi, Abbas Pardakhti, Ebrahim Ghaderi, Hasan Soltani, Sedigh Jadidoleslami, Anoush Arianejad, Hamed Gavili, Borhan Moradveisi, Dina Motamedi, Hamed Zare, Toba Kazemi, Mohammad Hassan Emamian

**Affiliations:** a School of Public Health, Shahroud University of Medical Sciences, Shahroud, Islamic Republic of Iran.; b Neuroscience Research Center, Mashhad University of Medical Sciences, Mashhad, Islamic Republic of Iran.; c Faculty of Health, Kurdistan University of Medical Sciences, Sanandaj, Islamic Republic of Iran.; d Ophthalmic Epidemiology Research Center, 7 Tir Square, Shahroud University of Medical Sciences, Shahroud 3614773947, Islamic Republic of Iran.; e Cellular and Molecular Research Center, Birjand University of Medical Sciences, Birjand, Islamic Republic of Iran.; f Social Determinants of Health Research Center, Gonabad University of Medical Sciences, Gonabad, Islamic Republic of Iran.; g Department of Epidemiology, Iran University of Medical Sciences, Tehran, Islamic Republic of Iran.; h National Institute for Health Research, Tehran University of Medical Sciences, Tehran, Islamic Republic of Iran.; i School of Computer Mathematics and Natural Sciences, Morgan State University, Baltimore, United States of America.; j Center for Health Related Social and Behavioral Sciences Research, Shahroud University of Medical Sciences, Shahroud, Islamic Republic of Iran.; k Health Technology Incubator Center, Shahroud University of Medical Sciences, Shahroud, Islamic Republic of Iran.; l Bahar Clinical Research Development Unit, Shahroud University of Medical Sciences, Shahroud, Islamic Republic of Iran.; m Razi Faculty of Nursing and Midwifery, Kerman University of Medical Sciences, Kerman, Islamic Republic of Iran.; n Health Promotion Research Center, Zahedan University of Medical Sciences, Zahedan, Islamic Republic of Iran.; o School of Medicine, Guilan University of Medical Sciences, Rasht, Islamic Republic of Iran.; p Infectious Diseases and Tropical Medicine Research Center, Zahedan University of Medical Sciences, Zahedan, Islamic Republic of Iran.; q Vice-Chancellery for Health Affairs, Guilan University of Medical Sciences, Rasht, Islamic Republic of Iran.; r Imam Hossein Clinical Research Development Unit, Shahroud University of Medical Sciences, Shahroud, Islamic Republic of Iran.; s School of Allied Medical Sciences, Shahroud University of Medical Sciences, Shahroud, Islamic Republic of Iran.; t Department of Infectious Diseases and Tropical Medicine, Mashhad University of Medical Sciences, Mashhad, Islamic Republic of Iran.; u Lung Diseases Research Center, Mashhad University of Medical Sciences, Mashhad, Islamic Republic of Iran.; v Allergy Research Center, Mashhad University of Medical Sciences, Mashhad, Islamic Republic of Iran.; w Antimicrobial Resistance Research Center, Mashhad University of Medical Sciences, Mashhad, Islamic Republic of Iran.; x Department of Cardiology, Mashhad University of Medical Sciences, Mashhad, Islamic Republic of Iran.; y HIV/STI Surveillance Research Center, Kerman University of Medical Sciences, Kerman, Islamic Republic of Iran.; z Department of Internal Medicine, Kerman University of Medical Sciences, Kerman, Islamic Republic of Iran.; aa Cardiovascular Research Centre, Kerman University of Medical Sciences, Kerman, Islamic Republic of Iran.; ab Department of Immunology, Kerman University of Medical Sciences, Kerman, Islamic Republic of Iran.; ac Research Center for Hydatid Disease, Kerman University of Medical Sciences, Kerman, Islamic Republic of Iran.; ad Department of Medical Microbiology (Bacteriology & Virology), Kerman University of Medical Sciences, Kerman, Islamic Republic of Iran.; ae Pharmaceutics Research Center, Kerman University of Medical Sciences, Kerman, Islamic Republic of Iran.; af Zoonoses Research Center, Kurdistan University of Medical Science, Sanandaj, Islamic Republic of Iran.; ag Vice-Chancellery for Health Affairs, Kurdistan University of Medical Sciences, Sanandaj, Islamic Republic of Iran.; ah Vice-Chancellery for Clinical Affairs, Kurdistan University of Medical Sciences, Sanandaj, Islamic Republic of Iran.; ai Department of Pediatrics, Kurdistan University of Medical Sciences, Sanandaj, Islamic Republic of Iran.; aj Department of Neurology, Kurdistan University of Medical Sciences, Sanandaj, Islamic Republic of Iran.; ak Cardiovascular Diseases Research Center, Birjand University of Medical Sciences, Birjand, Islamic Republic of Iran.

## Abstract

**Objective:**

To investigate the incidence of coronavirus disease 2019 (COVID-19) cases, hospitalizations and deaths in Iranians vaccinated with either AZD1222 Vaxzevria, CovIran® vaccine, SARS-CoV-2 Vaccine (Vero Cell), Inactivated (lnCoV) or Sputnik V.

**Methods:**

We enrolled individuals 18 years or older receiving their first COVID-19 vaccine dose between April 2021 and January 2022 in seven Iranian cities. Participants completed weekly follow-up surveys for 17 weeks (25 weeks for AZD1222) to report their COVID-19 status and hospitalization. We used Cox regression models to assess risk factors for contracting COVID-19, hospitalization and death.

**Findings:**

Of 89 783 participants enrolled, incidence rates per 1 000 000 person-days were: 528.2 (95% confidence interval, CI: 514.0–542.7) for contracting COVID-19; 55.8 (95% CI: 51.4–60.5) for hospitalization; and 4.1 (95% CI: 3.0–5.5) for death. Compared with SARS-CoV-2 Vaccine (Vero Cell), hazard ratios (HR) for contracting COVID-19 were: 0.70 (95% CI: 0.61−0.80) with AZD1222; 0.73 (95% CI: 0.62–0.86) with Sputnik V; and 0.73 (95% CI: 0.63–0.86) with CovIran®. For hospitalization and death, all vaccines provided similar protection 14 days after the second dose. History of COVID-19 protected against contracting COVID-19 again (HR: 0.76; 95% CI: 0.69–0.84). Diabetes and respiratory, cardiac and renal disease were associated with higher risks of contracting COVID-19 after vaccination.

**Conclusion:**

The rates of contracting COVID-19 after vaccination were relatively high. SARS-CoV-2 Vaccine (Vero Cell) provided lower protection against COVID-19 than other vaccines. People with comorbidities had higher risks of contracting COVID-19 and hospitalization and should be prioritized for preventive interventions.

## Résumé

**Objectif:**

Analyser l'incidence des cas, hospitalisations et décès liés à la maladie à coronavirus 2019 (COVID-19) chez les Iraniens vaccinés avec Vaxzevria AZD1222, CovIran®, le vaccin contre le SARS-CoV-2 (cellule Vero), le vaccin inactivé (lnCoV) ou Spoutnik V.

**Méthodes:**

Nous avons recruté des individus de minimum 18 ans ayant reçu leur première dose de vaccin contre la COVID-19 entre avril 2021 et janvier 2022 dans sept villes iraniennes. Les participants ont rempli des enquêtes de suivi hebdomadaires pendant 17 semaines (25 semaines pour AZD1222) afin de communiquer sur leur statut COVID-19 et leur hospitalisation. Nous avons utilisé des modèles de régression de Cox pour évaluer les facteurs augmentant les risques de contracter la COVID-19, d'être hospitalisé et de mourir.

**Résultats:**

Sur 89 783 participants recrutés, le taux d'incidence pour 1 000 000 jours-personnes était de 528,2 (intervalle de confiance de 95%, IC: 514,0–542,7) en termes d'infection à la COVID-19; de 55,8 (IC de 95%: 51,4–60,5) en termes d'hospitalisation; et de 4,1 (IC de 95%: 3,0–5,5) en termes de décès. Comparé au vaccin contre le SARS-CoV-2 (cellule Vero), le rapport de risque (RR) de contracter la COVID-19 s'élevait à 0,70 (IC de 95%: 0,61−0,80) avec AZD1222; à 0,73 (IC de 95%: 0,62–0,86) avec Spoutnik V; et à 0,73 (IC de 95%: 0,63–0,86) avec CovIran®. Concernant l'hospitalisation et le décès, tous les vaccins offraient un niveau de protection similaire 14 jours après la seconde dose. Avoir contracté la COVID-19 par le passé prémunissait contre une réinfection (RR: 0,76; IC de 95%: 0,69–0,84). Le diabète et les maladies respiratoires, cardiaques et rénales entraînaient un risque accru de contracter la COVID-19 après la vaccination.

**Conclusion:**

Le taux d'infection à la COVID-19 après la vaccination était relativement élevé. D'autre part, le vaccin contre le SARS-CoV-2 (cellule Vero) s'est révélé moins efficace que les autres vaccins. Enfin, les personnes souffrant de comorbidités avaient plus de risques de contracter la COVID-19 et d'être hospitalisées; elles devraient donc être prioritaires lors des interventions préventives.

## Resumen

**Objetivo:**

Analizar la incidencia de los casos, hospitalizaciones y muertes a causa de la enfermedad por coronavirus de 2019 (COVID-19) en los iraníes vacunados con Vaxzevria AZD1222, la vacuna CovIran®, la vacuna contra el SARS-CoV-2 (célula Vero), la vacuna inactivada (lnCoV) o la Sputnik V.

**Métodos:**

Se inscribieron personas de 18 años o más que recibieron su primera dosis de la vacuna contra la COVID-19 entre abril de 2021 y enero de 2022 en siete ciudades iraníes. Los participantes completaron encuestas de seguimiento semanales durante 17 semanas (25 semanas para AZD1222) para informar sobre su estado de la COVID-19 y la hospitalización. Se utilizaron modelos de regresión de Cox para valorar los factores de riesgo de contraer la COVID-19, de ser hospitalizado y de morir.

**Resultados:**

De los 89 783 participantes inscritos, las tasas de incidencia por 1 000 000 de días-persona fueron 528,2 (intervalo de confianza del 95 %, IC: 514,0-542,7) en el caso de contraer la COVID-19; 55,8 (IC del 95 %: 51,4-60,5) en el caso de hospitalización; y 4,1 (IC del 95 %: 3,0-5,5) en el caso de muerte. En comparación con la vacuna contra el SARS-CoV-2 (célula Vero), los cocientes de riesgos instantáneos (CRI) de contraer la COVID-19 fueron 0,70 (IC 95 %: 0,61-0,80) con AZD1222; 0,73 (IC 95 %: 0,62-0,86) con Sputnik V; y 0,73 (IC 95 %: 0,63-0,86) con CovIran®. En cuanto a la hospitalización y la muerte, todas las vacunas proporcionaron una protección similar 14 días después de la segunda dosis. Los antecedentes de la COVID-19 protegieron contra la contracción de la COVID-19 de nuevo (CRI: 0,76; IC 95 %: 0,69-0,84). La diabetes y las enfermedades respiratorias, cardíacas y renales se asociaron a un mayor riesgo de contraer la COVID-19 después de la vacunación.

**Conclusión:**

Las tasas de contraer la COVID-19 después de la vacunación fueron relativamente altas. La vacuna contra el SARS-CoV-2 (célula Vero) proporcionó una menor protección contra la COVID-19 que otras vacunas. Las personas con comorbilidades tenían un mayor riesgo de contraer la COVID-19 y de ser hospitalizadas, por lo que deberían ser prioritarias para las intervenciones preventivas.

## ملخص

**الغرض:**

التحقيق في حالات الإصابة بمرض فيروس كورونا 2019 (كوفيد 19)، وحالات الاستشفاء والوفيات بين الإيرانيين الذين تم تطعيمهم إما باستخدام لقاح AZD1222 Vaxzevria، أو لقاح CovIran®‎، أو لقاح SARS-CoV-2 )Vero Cell)، أو لقاحي (lnCoV) أو Sputnik V غير النشطين.

**الطريقة:**

قمنا بتسجيل الأفراد الذين تبلغ أعمارهم 18 عامًا أو أكثر، والذين تلقوا أول جرعة لقاح لكوفيد 19 بين أبريل/نيسان 2021 ويناير/كانون الثاني 2022، في سبع مدن إيرانية. أكمل المشاركون استطلاعات المتابعة الأسبوعية لمدة 17 أسبوعًا (25 أسبوعًا بالنسبة للقاح AZD1222) للإبلاغ عن تطور حالة كوفيد 19 والاستشفاء. استخدمنا نماذج التحوف كوكس (Cox) لتقييم عوامل الخطر للإصابة بكوفيد 19، والاستشفاء والوفاة.

**النتائج:**

من إجمالي 89783 مشاركًا تم تسجيلهم، فإن معدلات حدوث الإصابة لكل 1000000 شخص-يوم، كانت: 528.2 (بفاصل ثقة مقداره %95: 514.0 إلى 542.7) للإصابة بكوفيد 19؛ و55.8 (بفاصل ثقة مقداره %95: 51.4 إلى 60.5) لحالات الاستشفاء؛ و4.1 (بفاصل ثقة مقداره %95: 3.0 إلى 5.5) للوفيات. بالمقارنة مع لقاح SARS-CoV-2 Vaccine )Vero Cell)، كانت نسب المخاطرة (HR) للإصابة بكوفيد 19: 0.70 (بفاصل ثقة مقداره %95: 0.61 إلى 0.80) مع AZD1222؛ و0.73 (بفاصل ثقة مقداره%95: 0.62 إلى 0.86) مع Sputnik V؛ و0.73 (بفاصل ثقة مقداره %95: 0.63 إلى 0.86) مع CovIran®‎. بالنسبة لحالات الاستشفاء والوفيات، فقد قدمت جميع اللقاحات حماية مماثلة بعد 14 يومًا من الجرعة الثانية. تاريخ الحماية من كوفيد 19 في مقابل الإصابة بكوفيد 19 مرة أخرى (نسب المخاطرة: 0.76؛ ‏بفاصل ثقة مقداره %95: 0.69 إلى 0.84). ارتبط مرض السكري، وأمراض الجهاز التنفسي، والقلب، والكلى بمخاطر أعلى للإصابة بكوفيد 19 بعد التطعيم.

**الاستنتاج:**

كانت معدلات الإصابة بكوفيد 19 بعد التطعيم مرتفعة نسبيًا. وفّر لقاح SARS-CoV-2 )Vero Cell) حماية أقل ضد الإصابة بكوفيد 19 مقارنةً بالتطعيمات الأخرى. عاني الأشخاص المصابون بأمراض مشتركة من مخاطر أعلى للإصابة بكوفيد 19 والاستشفاء، ويجب أن يحصلوا على الأولوية في التدخلات الوقائية.

## 摘要

**目的:**

调查接种 AZD1222 Vaxzevria、CovIran® 疫苗、SARS-CoV-2 疫苗（Vero Cell）、灭活疫苗（lnCoV）或 Sputnik V 疫苗的伊朗人感染新型冠状病毒肺炎 (COVID-19) 的情况及其住院率和死亡率。

**方法:**

我们在七个伊朗城市招募了 2021 年 4 月至 2022 年 1 月之间接种第一剂新冠疫苗的 18 岁或以上的人员。参与者完成了为期 17 周（AZD1222 为 25 周）的每周跟进调查，报告他们的新冠肺炎状况和住院情况。我们使用 Cox 回归模型来评估感染新冠肺炎、住院和死亡的风险因子。

**结果:**

在招募的 89,783 名参与者中，每百万人每天感染新冠肺炎的比例为：528.2（95％ 置信区间，CI: 514.0-542.7)；住院比例为 55.8 (95% CI：51.4-60.5）；死亡比例为 4.1 (95% CI: 3.0–5.5)。与 SARS-CoV-2 疫苗（Vero Cell）相比，接种 AZD1222 疫苗后感染新冠肺炎的风险比（HR）为：0.70 (95% CI：0.61-0.80)；接种 Sputnik V 疫苗后感染的风险比为 0.73 (95% CI：0.62-0.86）；接种 CovIran® 疫苗后感染的风险比为 0.73 (95% CI: 0.63–0.86)。对于住院和死亡病例，所有疫苗在接种第二剂后的 14 天内均提供相同程度的保护力。感染过新冠肺炎可以避免再次感染（HR：0.76；95% CI：0.69-0.84)。患有糖尿病、呼吸系统、心脏和肾脏疾病的人群在接种疫苗后感染新冠肺炎的风险更高。

**结论:**

接种疫苗后感染新冠肺炎的比例较高。与其他疫苗相比，SARS-CoV-2 疫苗（Vero Cell）防止感染新冠肺炎的效果较低。并存其它疾病的患者感染新冠肺炎和住院的风险较高，应优先采取预防性干预措施。

## Резюме

**Цель:**

Изучить уровень заболеваемости коронавирусной инфекцией 2019 года (COVID-19), случаи госпитализации и смерти среди иранцев, вакцинированных вакциной AZD1222 Vaxzevria, CovIran®, SARS-CoV-2 (Vero Cell), инактивированной вакциной (lnCoV) или вакциной Sputnik V.

**Методы:**

Авторы включили в исследование участников в возрасте от 18 лет, получивших первую дозу вакцины против COVID-19 в период с апреля 2021 г. по январь 2022 г. в семи иранских городах. Участники проходили еженедельные последующие обследования в течение 17 недель (25 недель для AZD1222) и сообщали о своем состоянии COVID-19 и госпитализации. Авторы использовали модели регрессии Кокса для оценки факторов риска заражения COVID-19, госпитализации и смерти.

**Результаты:**

Из 89 783 включенных участников показатели заболеваемости на 1 000 000 человеко-дней составили: 528,2 (95%-й ДИ: 514,0–542,7) для случаев заражения COVID-19; 55,8 (95%-й ДИ: 51,4–60,5) для случаев госпитализации; 4,1 (95%-й ДИ: 3,0–5,5) для случаев смерти. По сравнению с вакциной SARS-CoV-2 (Vero Cell) коэффициенты риска (HR) для заражения COVID-19 были следующими: 0,70 (95%-й ДИ: 0,61–0,80) для AZD1222; 0,73 (95%-й ДИ: 0,62–0,86) для Sputnik V; 0,73 (95%-й ДИ: 0,63–0,86) для CovIran®. В отношении случаев госпитализации и смертей все вакцины обеспечивали одинаковую защиту через 14 дней после введения второй дозы. Наличие вакцины против COVID-19 защищает от повторного заражения COVID-19 (HR: 0,76; 95%-й ДИ: 0,69–0,84). Диабет, а также респираторные, сердечные и почечные заболевания были связаны с более высоким риском заражения COVID-19 после вакцинации.

**Вывод:**

Уровень заражения COVID-19 после вакцинации был относительно высоким. Вакцина против SARS-CoV-2 (Vero Cell) обеспечивает более низкую защиту от COVID-19, чем другие вакцины. Люди с сопутствующими заболеваниями имели более высокий риск заражения COVID-19 и госпитализации, поэтому профилактические вмешательства для них следует осуществлять в приоритетном порядке.

## Introduction

To end the ongoing global pandemic of coronavirus disease 2019 (COVID-19), it is imperative to have safe and effective vaccines that can provide immunity against severe acute respiratory syndrome coronavirus 2 (SARS-CoV-2) infection. As of 10 June 2022, 364 vaccines against SARS-CoV-2 have been investigated, but only 37 of them have been used in Phase III clinical trials.^1^

New vaccines are developed by companies and research centres using a broad range of techniques including viral vectors, inactivated vaccines, live weakened vaccines, deoxyribonucleic acid- (DNA) or ribonucleic acid- (RNA) based vaccines and protein-based vaccines.^2^^,^^3^ Each technique has advantages and disadvantages. For example, viral vector vaccines trigger robust immune responses that can result in long-term protection;^4^ however, they may also cause serious complications, such as immunity against the vector and coagulopathy.^5^ Inactivated vaccines, on the other hand, while safe for immunocompromised individuals,^6^ usually induce a much weaker immune response than viral vector-based vaccines.^7^

As of 12 January 2022, the World Health Organization (WHO) lists only nine COVID-19 vaccines that have been deemed safe and efficacious for emergency use in national immunization programmes.^8^ The approvals, however, are based on evidence from randomized controlled clinical trials whose samples may not necessarily be representative of the general population. Furthermore, only interim analyses were done for licensing purposes, the data did not allow the duration of protection to be determined and certain populations, such as pregnant women, were excluded. Therefore, active surveillance of the vaccines is needed through observational studies on the incidence of adverse events and COVID-19 cases and hospitalizations among vaccinated individuals over a defined period of time. To conduct an active safety surveillance study, a protocol template for cohort event monitoring studies has been released by WHO.^9^

To meet the need for such data in the Islamic Republic of Iran for four COVID-19 vaccines (i.e. AZD1222 Vaxzevria, CovIran® vaccine, SARS-CoV-2 Vaccine (Vero Cell), Inactivated (lnCoV) or Sputnik V), we used the WHO protocol template for cohort event monitoring to design an observational study on the incidence of serious adverse events, adverse events of special interest and COVID-19 after each vaccine dose. We also sought to estimate the reactogenicity within 7 days of receiving each vaccine dose.^10^ In this paper, we report the incidence of COVID-19 cases, hospitalizations and deaths among a vaccinated sample of the population. It should be noted that the objective of this study was to determine the safety and efficiency of each vaccine in terms of adverse events and COVID-19 and is in no way related to WHO emergency approval, nor does it reflect the opinions of WHO.

## Methods

In accordance with the cohort event monitoring template developed by WHO, we conducted a four-arm cohort study in seven cities in the Islamic Republic of Iran (Birjand, Kerman, Mashhad, Rasht, Sanandaj, Shahroud and Zahedan). The selection of the study sites was based on availability of tertiary care hospitals and an experienced investigator and also on the commitment of local authorities to support the study. We invited individuals 18 years or older who were receiving their first dose of SARS-CoV-2 Vaccine (Vero Cell), Inactivated (lnCoV), Sputnik V, AZD1222 Vaxzevria or CovIran® vaccine at a participating public vaccination site to participate. This study is part of a larger study,^10^ and here we report the incidence of COVID-19 cases, hospitalizations for COVID-19 and deaths due to COVID-19 by vaccine brand. The study methods have been fully described previously^10^ and we provide a summary as follows.

After enrolment, study staff interviewed the participants and collected their contact information and other data on vaccine brand, vaccination date, vaccine batch number, demographic characteristics (age, sex, years of education), previous COVID-19 and comorbidities (cancer, chronic cardiac, hepatic, mental, neurological, respiratory and renal diseases, diabetes, hypertension and immunodeficiency). To assess obesity, we also recorded self-reported weight and height and defined obesity as a body mass index ≥ 30 kg/m^2^. We actively followed participants until 3 months after their last dose of COVID-19 vaccine, if administered within 3 months of the first dose. For those receiving SARS-CoV-2 Vaccine (Vero Cell), Inactivated (lnCoV), Sputnik V or CovIran® vaccine, the expected follow-up time was 17 weeks, assuming an interval of up to 1 month between the first and second doses. For those receiving AZD1222 Vaxzevria, given that the interval between doses is 3 months, the expected follow-up time was 25 weeks.

During the follow-up period, participants completed questionnaires through telephone or web-based surveys at weekly intervals. We also retrieved vaccination dates and vaccine batch numbers of the second doses from national COVID-19 vaccination registries. The diagnosis of COVID-19 infection was based on self-reported reverse-transcription polymerase chain reaction (PCR) test or antibodies against SARS-CoV-2. We also checked medical hospital records for unreliable responses (i.e. if the participant did not know the PCR results) and for patients admitted to intensive care units.

We considered participants lost to follow-up after two unsuccessful attempts to contact them by telephone, followed by one unsuccessful attempt to contact their next of kin. In case of loss to follow-up, we used data collected up to the last follow-up time in the analyses. We calculated the follow-up index (ratio) as the: actual investigated follow-up period/potential follow-up duration.^11^

To factor in immunity status (time elapsed since vaccine dose administration), we considered the following immunity periods: non-immune period = first 14 days after the administration of the first dose; partial immunity period = period between 14 days after the first dose and 14 days after the second dose; full immunity period = period between 14 days after the second dose and the end of follow-up. We calculated incidence rates by dividing the total number of events by total person-days followed up, with their 95% confidence intervals (CI), for the total sample and by age, sex and immunity status. For the total follow-up period, we calculated incidence rates for events occurring 14 days after the first dose of the vaccine.

We used Cox proportional hazard regression models for survival analysis, with calendar time as the timescale and stratified by study sites to estimate the adjusted hazard ratios (HRs) for COVID-19 infection. We first used a univariate model with age, sex, education, vaccine brand, prior COVID-19 and comorbidities as covariates, and we only entered variables significant at *P* < 0.1 into the final stepwise Cox regression models.

### Ethical considerations

The Institutional Review Board of Shahroud University of Medical Sciences, Islamic Republic of Iran (IR.SHMU.REC.1400.012) approved the study protocol, and we conducted all procedures in accordance with the Helsinki Declaration. Study participation was voluntary and all participants gave their written informed consent after trained staff had explained the study objectives and procedures and had answered participants’ questions.

## Results

**Fig. 1:**
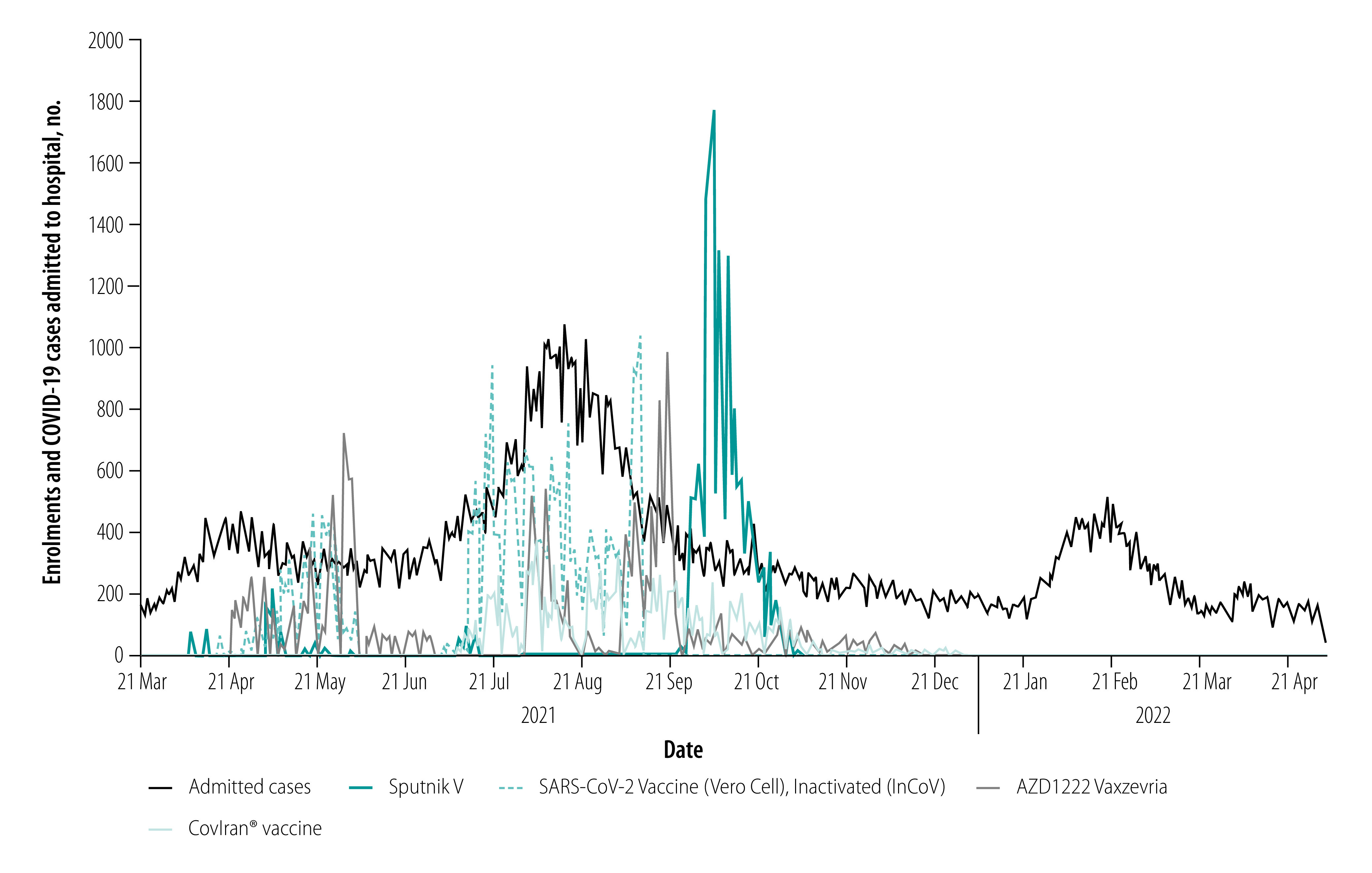
Daily enrolments in the study by vaccine brand and COVID-19 cases admitted to hospital, Islamic Republic of Iran, 21 March 2021–2 May 2022

Between 7 April 2021 and 22 January 2022, we enrolled 89 783 participants in the study (Fig. 1). Table 1 presents the distribution of participants by vaccine brand and vaccination status, demographic characteristics, underlying diseases and follow-up status. The follow-up index was more than 98% for all vaccines.

COVID-19: coronavirus disease 2019.

**Table 1:** Characteristics of study participants by vaccine brand, Islamic Republic of Iran, 2021

Variable	SARS-CoV-2 Vaccine (Vero Cell), Inactivated (lnCoV)	Sputnik V	AZD1222 Vaxzevria	CovIran® vaccine
**Total, no. (%)**	31 690 (35.3)	20 195 (22.5)	23 780 (26.5)	14 118 (15.7)
**Enrolment start date**	17 Apr 2021	7 Apr 2021	21 Apr 2021	5 Jul 2021
**Date of completion of vaccination^a^**				
First quartile	15 Aug 2021	1 Nov 2021	5 Sep 2021	12 Sep 2021
Second quartile	4 Sep 2021	8 Nov 2021	4 Nov 2021	13 Oct 2021
Third quartile	25 Sep 2021	22 Nov 2021	16 Dec 2021	16 Nov 2021
**Received second dose, no. (%)**	30 228 (95.4)	17 701 (87.7)	21 379 (89.9)	12 499 (88.5)
**Received third dose, no. (%)**	490 (1.5)	105 (0.52)	22 (0.1)	64 (0.5)
**Age in years, mean (SD)**	53.3 (16.0)	34.4 (11.1)	45.6 (18.3)	42.8 (13.2)
**Males, no. (%)**	15 535 (49.0)	11 129 (55.1)	12 923 (54.3)	7411 (52.5)
**Education in years, mean (SD)**	10.9 (5.4)	13.3 (3.5)	12.3 (5.2)	12.1 (4.3)
**Medical history, no. (%)**				
Prior COVID-19 infection (self-reported)	7062 (22.3)	8549 (42.3)	6100 (25.7)	4205 (29.8)
Obesity	3738 (11.8)	2158 (10.7)	2576 (10.8)	1384 (9.8)
Diabetes	4275 (13.5)	339 (1.7)	1981 (8.3)	981 (7.0)
Hypertension	5993 (18.9)	526 (2.6)	2988 (12.6)	1136 (8.1)
Chronic cardiac diseases	2962 (9.4)	242 (1.2)	1499 (6.3)	515 (3.7)
Cancer	1007 (3.2)	19 (0.1)	75 (0.3)	30 (0.2)
Chronic respiratory diseases	861 (2.7)	188 (0.9)	446 (1.9)	230 (1.6)
Chronic renal diseases	601 (1.9)	64 (0.3)	255 (1.1)	128 (0.9)
Chronic hepatic diseases	260 (0.8)	52 (0.3)	139 (0.6)	82 (0.6)
Chronic neurological diseases	402 (1.3)	79 (0.4)	193 (0.8)	156 (1.1)
Mental health disorders	146 (0.5)	30 (0.2)	107 (0.5)	55 (0.4)
Immunodeficiency	64 (0.2)	19 (0.1)	30 (0.1)	12 (0.1)
**Follow-up index, %^b^**	98.4	98.6	99.4	98.6

COVID-19: coronavirus disease 2019; SD: standard deviation.

^a^ The first quartile date is when a quarter of the participants received their second dose of vaccine; the second quartile date is when half of the participants received their second dose of vaccine; the third quartile date is when three quarters of the participants received their second dose of vaccine.

^b^ We calculated the follow-up index (ratio) as the: actual investigated follow-up period/potential follow-up duration.^11^

The incidence rate of COVID-19 cases was 528.2 (95% CI: 514.0–542.7) per 1 000 000 person-days. For hospitalizations and deaths incidence rates were 55.8 (95% CI: 51.4–60.5) and 4.1 (95% CI: 3.0–5.5) cases per 1 000 000 person-days, respectively. Table 2 shows these rates for the different vaccine brands by sex, age and immunity status. Among the vaccine brands, the SARS-CoV-2 Vaccine (Vero Cell), Inactivated (lnCoV) had the highest rates of COVID-19 cases, hospitalizations and deaths. Participants 50 years and older had significantly higher rates of hospitalization and death compared with those younger than 50 years regardless of vaccine brand. In addition, with all vaccine brands, both hospitalization and death rates were significantly higher during the partial immunity period than the full immunity period.

**Table 2:** Incidence of COVID-19 infection, hospitalization and death by vaccine brand in the total sample and by subgroup, Islamic Republic of Iran, 2021

Outcome variable	Incidence, cases per 1 000 000 person-days (95% CI)				
	SARS-CoV-2 Vaccine (Vero Cell), Inactivated (lnCoV)	Sputnik V	AZD1222 Vaxzevria	CovIran® vaccine
**COVID-19 case**					
Total	613.3 (586.4–641.5)	328.5 (304.2–354.6)	595.4 (570.2–621.7)	456.3 (421.9–493.5)	
Males	595.8 (558.3–635.9)	280.4 (250.7–313.6)	536.5 (504.3–570.7)	463.7 (416.6–616.2)	
Females	630.2 (592.2–670.6)	387.2 (348.5–430.2)	665.1 (626.1–706.6)	448.2 (399.6–502.6)	
18–49 years	561.6 (522.9–603.2)	330.5 (305.2–358.0)	668.3 (635.2–703.1)	433.5 (393.8–477.2)	
≥ 50 years	652.8 (616.1–691.7)	305.6 (231.6–403.2)	462.4 (425.8–502.1)	509.5 (445.1–583.3)	
Partial immunity^a^	870.6 (816.4–928.5)	307.5 (271.0–349.0)	537.7 (506.7–570.7)	639.0 (574.4–710.9)	
Full immunity^b^	478.7 (449.5–509.7)	342.0 (310.6–376.6)	676.7 (635.4–720.7)	341.7 (304.4–383.5)	
**COVID-19 hospitalization**					
Total	98.4 (88.1–109.8)	21.8 (16.2–29.3)	40.1 (30.0–47.2)	47.5 (37.4–60.3)	
Males	98.4 (84.0–115.1)	26.2 (18.2–37.7)	38.1 (30.3–47.8)	47.2 (33.9–65.7)	
Females	98.4 (84.3–114.8)	16.5 (9.9–27.3)	42.4 (33.5–53.6)	47.8 (33.8–67.6)	
18–49 years	63.2 (51.3–77.8)	17.8 (12.7–25.1)	24.9 (19.3–32.2)	29.5 (20.5–42.4)	
≥ 50 years	125.3 (110.1–142.7)	66.4 (36.8–119.9)	67.9 (54.9–83.8)	89.0 (64.7–122.3)	
Partial immunity^a^	153.2 (131.8–178.1)	37.8 (26.5–54.1)	51.8 (42.9–62.6)	76.5 (56.5–103.5)	
Full immunity^b^	69.4 (59.0–81.7)	11.4 (6.8–19.3)	23.8 (17.2–33.0)	29.0 (19.6–42.9)	
**COVID-19 death**					
Total	7.7 (5.2–11.5)	0.5 (0.1–3.5)	3.0 (1.7–5.5)	3.5 (1.5–8.5)	
Males	9.5 (5.7–15.7)	0.9 (0.1–6.4)	3.1 (1.4–6.8)	4.0 (1.3–12.5)	
Females	6.1 (3.3–11.3)	0.0	3.0 (1.3–7.3)	4.0 (1.3–12.5)	
18–49 years	1.4 (0.4–5.7)	0	0.4 (0.1–3.0)	0	
≥ 50 years	12.6 (8.3–18.9)	6.0 (0.9–42.7)	7.9 (4.2–14.6)	11.6 (4.9–28.0)	
Partial immunity^a^	11.6 (6.8–20.0)	1.3 (0.2–8.9)	3.8 (1.9–7.7)	5.4 (1.8–16.9)	
Full immunity^b^	5.7 (3.2–10.0)	0.0	2.0 (0.6–6.1)	2.3 (0.6–9.3)	

CI: confidence interval; COVID-19: coronavirus disease 2019.

^a^ Defined as the period between the 14th day after the first dose and 14 days after the second dose.

^b^ Defined as the period between the 14th day after the second dose and the end of follow-up.

The results of Cox proportional hazard regression for COVID-19 infection are shown in Table 3. Sputnik V and AZD1222 Vaxzevria provided significantly greater protection than the SARS-CoV-2 Vaccine (Vero Cell), Inactivated (lnCoV) during the partial immunity period. In the full immunity period, the SARS-CoV-2 Vaccine (Vero Cell), Inactivated (lnCoV) provided lower protection than the other three vaccines. History of COVID-19 significantly reduced the risk of reinfection, and most underlying diseases significantly increased the risk of COVID-19, including chronic neurological disorders and mental health disorders.

**Table 3:** Variables associated with COVID-19 cases: Cox regression analysis, Islamic Republic of Iran, 2021

Independent variable	HR (95% CI)	
	Partial immunity period^a^	Full immunity period^b^
**Age, years**	1.00 (0.99–1.00)	1.00 (0.99–1.01)
**Female sex**	1.17 (1.08–1.27)	1.22 (1.13–1.32)
**Education, years**	1.02 (1.01–1.03)	1.05 (1.04–1.06)
**Vaccine brand**		
SARS-CoV-2 Vaccine (Vero Cell), Inactivated (lnCoV)	Reference	Reference
Sputnik V	0.66 (0.55–0.80)	0.73 (0.62–0.86)
AZD1222 Vaxzevria	0.74 (0.67–0.82)	0.70 (0.61−0.80)
CovIran® vaccine	0.93 (0.81–1.07)	0.73 (0.63–0.86)
**Prior COVID-19 infection**	0.56 (0.50–0.64)	0.76 (0.69–0.84)
**Chronic respiratory diseases**	1.28 (1.01–1.63)	1.34 (1.03–1.75)
**Chronic renal diseases**	1.33 (0.98–1.81)	1.49 (1.07–2.07)
**Chronic neurological diseases**	1.44 (1.05–1.98)	NA^c^
**Diabetes**	1.12 (0.99–1.27)	1.17 (1.01–1.37)
**Chronic cardiac diseases**	NA^c^	1.25 (1.05–1.48)
**Mental health disorders**	1.82 (1.13–2.95)	NA^c^

CI: confidence interval; COVID-19: coronavirus disease 2019; HR: hazard ratio; NA: not applicable.

^a^ Defined as the period between the 14th day after the first dose and 14 days after the second dose.

^b^ Defined as the period between the 14th day after the second dose and the end of follow-up.

^c^ Not entered in the final stepwise Cox regression models as *P* ≥ 0.1 in the univariate analysis.

Table 4 shows the Cox proportional hazard regression for COVID-19 hospitalization. In the partial immunity period, AZD1222 Vaxzevria had significantly better effectiveness than the SARS-CoV-2 Vaccine (Vero Cell), Inactivated (lnCoV), while in the full immunity period, no significant differences were found between vaccine brands. History of COVID-19 reduced the risk of hospitalization only in the partial immunity period. Most underlying diseases significantly increased the risk of COVID-19 hospitalization in the partial immunity period.

**Table 4:** Variables associated with COVID-19 hospitalization: Cox regression analysis, Islamic Republic of Iran, 2021

Independent variable	HR (95% CI)	
	Partial immunity period^a^	Full immunity period^b^
**Age, years**	1.03 (1.02–1.04)	1.03 (1.02–1.04)
**Female sex**	1.02 (0.82–1.26)	0.98 (0.74–1.28)
**Education, years**	1.02 (0.997–1.047)	1.02 (0.99–1.05)
**Vaccine brand**		
SARS-CoV-2 Vaccine (Vero Cell), Inactivated (lnCoV)	Reference	Reference
Sputnik V	0.62 (0.38–1.00)	0.86 (0.46–1.59)
AZD1222 Vaxzevria	0.47 (0.36–0.61)	0.71 (0.48–1.08)
CovIran® vaccine	0.91 (0.62–1.32)	1.16 (0.73–1.84)
**Prior COVID-19 infection**	0.42 (0.29–0.62)	0.66 (0.44–1.00)
**Chronic respiratory diseases**	1.96 (1.21–3.19)	NA^c^
**Chronic renal diseases**	2.05 (1.14–3.68)	2.52 (1.33–4.80)
**Chronic cardiac diseases**	1.38 (1.00–1.92)	NA^c^
**Diabetes**	1.63 (1.23–2.16)	1.75 (1.25–2.44)
**Obesity**	1.60 (1.22–2.11)	NA^c^

CI: confidence interval; COVID-19: coronavirus disease 2019; HR: hazard ratio; NA: not applicable.

^a^ Defined as the period between the 14th day after the first dose and 14 days after the second dose.

^b^ Defined as the period between the 14th day after the second dose and the end of follow-up.

^c^ Not entered in the final stepwise Cox regression models as *P* ≥ 0.1 in the univariate analysis.

A total of 42 COVID-19-related deaths were registered; 17 (40.5%) occurred during the full immunity period (more than 14 days after administering the second dose). As such, the incidence of COVID-19 deaths per 1 000 000 person-days was 4.1 (95% CI: 3.0–5.5) overall (14 days after the first dose of vaccine till the end of follow-up) and 3.0 (95% CI: 1.9–4.8) during the full immunity period. The HR in the Cox regression model for COVID-19 death was not significantly different by vaccine brand in the full immunity period, but AZD1222 Vaxzevria provided better protection from death in the partial immunity period. Age (HR: 1.11; 95% CI: 1.04–1.17) and chronic renal diseases (HR: 5.13; 95% CI: 1.15–22.93) were associated with significantly higher risk of death (Table 5).

**Table 5:** Variables associated with death due to COVID-19: Cox regression analysis, Islamic Republic of Iran, 2021

Independent variable	HR (95% CI)	
	Partial immunity period^a^	Full immunity period^b^
**Age, years**	1.06 (1.01–1.11)	1.11 (1.04–1.17)
**Female sex**	0.59 (0.25–1.37)	0.88 (0.33–2.38)
**Education, years**	0.98 (0.90–1.07)	0.98 (0.88–1.09)
**Vaccine brand**		
SARS-CoV-2 Vaccine (Vero Cell), Inactivated (lnCoV)	Reference	Reference
Sputnik V	1.18 (0.11–13.01)	NA^c^
AZD1222 Vaxzevria	0.32 (0.13–0.83)	0.56 (0.12 – 2.64)
CovIran® vaccine	0.97 (0.25–3.74)	2.89 (0.43 – 19.44)
**Chronic renal diseases**	NA^d^	5.13 (1.15–22.93)

CI: confidence interval; COVID-19: coronavirus disease 2019; HR: hazard ratio in multiple Cox regression model.

^a^ Defined as the period between the 14th day after the first dose and 14 days after the second dose.

^b^ Defined as the period between the 14th day after the second dose and the end of follow-up.

^c^ No deaths occurred.

^d^ Not entered in the final stepwise Cox regression models as *P* ≥ 0.1 in the univariate analysis.

## Discussion

Although vaccines provide adequate protection against SARS-CoV-2, their effectiveness never reaches 100%, and this protection is expected to further decline as immunity wanes over time and new virus strains emerge.^12^ In fact, all COVID-19 vaccines have waning protection. However, vaccinated individuals who do become infected experience less severe symptoms and have much lower risk of hospitalization and death compared with unvaccinated people with similar risk factors.^13^ The results of our study showed overall breakthrough rates of 528.2, 55.8 and 4.1 per 1 000 000 person-days for COVID-19 cases, hospitalizations and deaths, respectively.

The main determinants of breakthrough rates were: time since vaccination; the genetic variant of SARS-CoV-2; comorbidities; age; waning immunity; level of community adherence to mitigation strategies; and epidemic severity.^14^ Therefore, it would be difficult to draw valid comparisons of breakthrough rates with other studies. For example, in Washington state in the United States of America, the rate of breakthrough infection among over 5 million fully vaccinated people increased from 1 per 5000 between 17 January and 21 August 2021, to 589 per 5000 between 17 January and 14 May 2022.^15^ In addition, the comparison of different vaccine brands in our study is misleading because the participants entered the study at different calendar times when the severity stage of the epidemic and the dominant variant were different. A higher rate of breakthrough infection has been reported for delta variants of SARS-CoV-2.^16^ The incidence of COVID-19 cases in fully vaccinated people was about 100 cases per 100 000 population in the United States during August to late November 2021 when the delta variant was the main variant.^17^ The incidence of deaths related to COVID-19 was 0.38 per 100 000 among the same group and over the same time. In addition, during the week of 1 May 2021, the median number of incident cases of COVID-19 in New York State among the vaccinated population was 2.4 cases per 100 000 person-days (range 0.7 to 6.8). Rates increased after the delta variant became the most prevalent circulating variant and reached 16.4 cases per 100 000 person-days (range 8.3 to 27.9) among vaccinated people.^18^

In our study, with 17 registered deaths (0.028% of admitted patients), the mortality rate in fully vaccinated people was 3.0 per 1 000 000 person-days; this rate is much higher than the rate reported in Massachusetts in the United States (0.01%)^19^ but lower than Minnesota (0.032%).^13^ Differences in age and COVID-19 epidemic patterns, virus variants, vaccine effectiveness and health-care utilization are the main reasons for differences between results and these factors should be noted when comparing study results.

The lower incidence rates of COVID-19 hospitalization and death in the full immunity period compared with the partial immunity period in our study are consistent with previous reports on the effectiveness of COVID-19 vaccines. For example, analysis of National Immunization Management Service and the Coronavirus Clinical Information Network in the United Kingdom of Great Britain and Northern Ireland showed that out of 40 000 patients with COVID-19 who were admitted to hospital, 84% had not been vaccinated, 13% had only received their first vaccine dose and 3% had received both doses.^20^^,^^21^ It should be noted that participants in our study were enrolled at different times on the epidemic curve. For example, the SARS-CoV-2 Vaccine (Vero Cell), Inactivated (lnCoV) became available just before the onset of the fifth COVID-19 wave, while CovIran® vaccine and SARS-CoV-2 Vaccine (Vero Cell), Inactivated (lnCoV) were the main brands used in the vaccination programme when COVID-19 incidence was at its peak in July 2021. Therefore, the occurrence of COVID-19 cases was affected by the time of entry of participants into the study. In addition, vaccines may lose their effectiveness over time as newer strains of the virus emerge. Even the age groups and comorbidities of participants varied by vaccine brands, and since the risk of infection differs by age and comorbidity, we cannot simply compare the incidence rates by vaccine brand. Thus, we used Cox regression analysis to adjust for important covariates and used calendar time as the time span.

Based on the results of the Cox regression analysis, AZD1222 Vaxzevria was most effective at preventing COVID-19 cases, hospitalizations and deaths, while the SARS-CoV-2 Vaccine (Vero Cell), Inactivated (lnCoV) had the lowest effectiveness, especially in the partial immunity period. A similar finding was recently reported in a large study conducted in the Islamic Republic of Iran.^22^ The results suggest that vector-based vaccines (Sputnik V and AZD1222 Vaxzevria) had better effectiveness than inactivated vaccines (SARS-CoV-2 Vaccine (Vero Cell), Inactivated (lnCoV) and CovIran® vaccine).

The Cox regression models showed that individuals with chronic respiratory, renal and cardiac diseases, diabetes and obesity were at a higher risk of COVID-19 hospitalization, and a previous history of COVID-19 reduced this risk to less than half. Similar associations have been reported in other studies.^23^^,^^24^ A study has shown that prior SARS-CoV-2 infection significantly reduces the risk of breakthrough infection.^25^ Another factor associated with COVID-19 hospitalization was obesity. This outcome is most likely because obesity impairs immunity by altering the response of cytokines which increases susceptibility to infection, especially infections that require a rapid cellular immune response.^26^^,^^27^ In addition, obesity is linked to metabolic disorders and other critical diseases such as diabetes, hypertension, and cardiac and cerebrovascular diseases. Obesity and its related comorbidities have been shown to increase the cumulative risk of death in COVID-19 patients.^24^^,^^28^ Similar to our findings, other studies confirmed the association of chronic neurological and mental diseases with contracting COVID-19.^29^ Higher vulnerability to SARS-CoV-2 infection of participants with mental diseases may be attributed to their lower cooperation with preventive measures, which may also be true for people with dementia and Parkinson disease. However, more studies are needed to determine the exact reasons for the association between mental and neurological diseases and SARS-CoV-2 infection.^29^

The main strengths of our study include its large sample size, the investigation and comparison of four vaccines, the active surveillance that was conducted, weekly follow-up of participants, and investigation and classification of all hospitalized participants. However, we could not determine virus variants and included no control group for investigating vaccine effectiveness, which can be considered limitations of our study.

In conclusion, COVID-19 breakthrough rates were relatively high in our study. AZD1222 Vaxzevria vaccine provided better protection from COVID-19 infection, hospitalization and death than the other three vaccines. All the vaccines had similar protection against COVID-19 hospitalization 14 days after the second dose. People with comorbidities had higher risk of contracting COVID-19 and hospitalization and should be prioritized for preventive interventions.

## References

[1] COVID-19 vaccine tracker and landscape, Geneva: World Health Organization; 2022. Available from: https://www.who.int/publications/m/item/draft-landscape-of-covid-19-candidate-vaccines [cited 2022 June 13].

[2] Tregoning JS, Brown ES, Cheeseman HM, Flight KE, Higham SL, Lemm NM, et al. Vaccines for COVID-19. Clin Exp Immunol. 2020 Nov;202(2):162–92. 10.1111/cei.13517 32935331 PMC7597597

[3] Thanh Le T, Andreadakis Z, Kumar A, Gómez Román R, Tollefsen S, Saville M, et al. The COVID-19 vaccine development landscape. Nat Rev Drug Discov. 2020 May;19(5):305–6. 10.1038/d41573-020-00073-5 32273591

[4] Barros-Martins J, Hammerschmidt SI, Cossmann A, Odak I, Stankov MV, Morillas Ramos G, et al. Immune responses against SARS-CoV-2 variants after heterologous and homologous ChAdOx1 nCoV-19/BNT162b2 vaccination. Nat Med. 2021 Sep;27(9):1525–9. 10.1038/s41591-021-01449-9 34262158 PMC8440184

[5] Monagle P, Ng AP, Linden M, Ignjatovic V, Farley A, Taoudi S, et al. Vaccine-induced immune thrombosis and thrombocytopenia syndrome following adenovirus-vectored severe acute respiratory syndrome coronavirus 2 vaccination: a novel hypothesis regarding mechanisms and implications for future vaccine development. Immunol Cell Biol. 2021 Nov;99(10):1006–10. 10.1111/imcb.12505 34664303 PMC8652900

[6] Lim WW, Mak L, Leung GM, Cowling BJ, Peiris M. Comparative immunogenicity of mRNA and inactivated vaccines against COVID-19. Lancet Microbe. 2021 Sep;2(9):e423. 10.1016/S2666-5247(21)00177-4 34308395 PMC8282488

[7] Iversen PL, Bavari S. Inactivated COVID-19 vaccines to make a global impact. Lancet Infect Dis. 2021 Jun;21(6):746–8. 10.1016/S1473-3099(21)00020-7 33548196 PMC7906657

[8] Coronavirus disease (COVID-19): vaccines [internet]. Geneva: World Health Organization; 2022. Available from: https://www.who.int/news-room/questions-and-answers/item/coronavirus-disease-(covid-19)-vaccines [cited 2022 May 18].

[9] Protocol template to be used as template for observational study protocols: cohort event monitoring (‎CEM)‎ for safety signal detection after vaccination with COVID-19 vaccines. Geneva: World Health Organization; 2021. Available from: https://apps.who.int/iris/handle/10665/342193 [cited 2022 May 18].

[10] Aliyari R, Mahdavi S, Enayatrad M, Sahab-Negah S, Nili S, Fereidooni M, et al. Study protocol: cohort event monitoring for safety signal detection after vaccination with COVID-19 vaccines in Iran. BMC Public Health. 2022;22(1):1153. 10.1186/s12889-022-13575-1 35681132 PMC9178529

[11] von Allmen RS, Weiss S, Tevaearai HT, Kuemmerli C, Tinner C, Carrel TP, et al. Completeness of follow-up determines validity of study findings: results of a prospective repeated measures cohort study. PLoS One. 2015 Oct 15;10(10):e0140817. 10.1371/journal.pone.0140817 26469346 PMC4607456

[12] Yewdell JW. Individuals cannot rely on COVID-19 herd immunity: durable immunity to viral disease is limited to viruses with obligate viremic spread. PLoS Pathog. 2021 Apr 26;17(4):e1009509. 10.1371/journal.ppat.1009509 33901246 PMC8075217

[13] COVID-19 vaccine breakthrough weekly update [internet]. St Paul: Minnesota Department of Health; 2022. Available from: https://www.health.state.mn.us/diseases/coronavirus/stats/vbt.html [cited 2022 May 10].

[14] Lipsitch M, Krammer F, Regev-Yochay G, Lustig Y, Balicer RD. SARS-CoV-2 breakthrough infections in vaccinated individuals: measurement, causes and impact. Nat Rev Immunol. 2022 Jan;22(1):57–65. 10.1038/s41577-021-00662-4 34876702 PMC8649989

[15] SARS-CoV-2 vaccine breakthrough surveillance and case information resource. Shoreline, WA: Washington State Department of Health; 2022. Available from: https://doh.wa.gov/sites/default/files/2022-02/420-339-VaccineBreakthroughReport.pdf [cited 2022 May 28].

[16] Christensen PA, Olsen RJ, Long SW, Subedi S, Davis JJ, Hodjat P, et al. Delta variants of SARS-CoV-2 cause significantly increased vaccine breakthrough COVID-19 cases in Houston, Texas. Am J Pathol. 2022 Feb;192(2):320–31. 10.1016/j.ajpath.2021.10.019 34774517 PMC8580569

[17] COVID data tracker. Rates of COVID-19 cases and deaths by vaccination status [internet]. Atlanta: Centers for Disease Control and Prevention; 2022. Available from: https://covid.cdc.gov/covid-data-tracker/#rates-by-vaccine-status [cited 2022 May 12].

[18] Rosenberg ES, Dorabawila V, Easton D, Bauer UE, Kumar J, Hoen R, et al. COVID-19 vaccine effectiveness in New York State. N Engl J Med. 2022 Jan 13;386(2):116–27. 10.1056/NEJMoa2116063 34942067 PMC8693697

[19] Breakthrough cases in Mass. Top 100,000; over 5 million fully vaccinated. NBC10 Boston. 2021 Dec 14. Available from: https://www.nbcboston.com/news/coronavirus/breakthrough-cases-in-mass-top-100000-over-5-million-fully-vaccinated/2590370/ [cited 2022 May 15].

[20] Egan C, Turtle L, Thorpe M, Harrison EM, Semple MG, Docherty AB; ISARIC4C Investigators. Hospital admission for symptomatic COVID-19 and impact of vaccination: analysis of linked data from the Coronavirus Clinical Information Network and the National Immunisation Management Service. Anaesthesia. 2022 Feb 18;77(5):605–8. 10.1111/anae.15677 35178709 PMC9111458

[21] Iacobucci G. Covid-19: how is vaccination affecting hospital admissions and deaths? BMJ. 2021 Sep 20;374(2306):n2306. 10.1136/bmj.n2306 34544731

[22] Taherian Z, Rezaei M, Haddadpour A, Amini Z. The effect of COVID-19 vaccination on reducing the risk of infection, hospitalization, and death in Isfahan Province, Iran. Iran J Public Health. 2022 Jan;51(1):188–95. 10.18502/ijph.v51i1.8311PMID:35223640 35223640 PMC8837898

[23] Jamali-Atergeleh H, Emamian MH, Goli S, Rohani-Rasaf M, Hashemi H, Fotouhi A. The risk factors of COVID-19 in 50–74 years old people: a longitudinal population-based study. Epidemiol Methods. 2021;10(s1): 20210024. 10.1515/em-2021-0024

[24] Poly TN, Islam MM, Yang HC, Lin MC, Jian WS, Hsu MH, et al. Obesity and mortality among patients diagnosed with COVID-19: a systematic review and meta-analysis. Front Med (Lausanne). 2021 Feb 5;8:620044. 10.3389/fmed.2021.620044 33634150 PMC7901910

[25] Abu-Raddad LJ, Chemaitelly H, Ayoub HH, Yassine HM, Benslimane FM, Al Khatib HA, et al. Association of prior SARS-CoV-2 infection with risk of breakthrough infection following mRNA vaccination in Qatar. JAMA. 2021 Nov 16;326(19):1930–9. 10.1001/jama.2021.19623 34724027 PMC8561432

[26] Rojas-Osornio SA, Cruz-Hernández TR, Drago-Serrano ME, Campos-Rodríguez R. Immunity to influenza: impact of obesity. Obes Res Clin Pract. 2019 Sep–Oct;13(5):419–29. 10.1016/j.orcp.2019.05.003 31542241

[27] Fortis A, García-Macedo R, Maldonado-Bernal C, Alarcón-Aguilar F, Cruz M. El papel de la inmunidad innata en la obesidad. Salud Publica Mex. 2012 Mar-Apr;54(2):171–7. Spanish. 10.1590/S0036-36342012000200014 22535177

[28] Mahamat-Saleh Y, Fiolet T, Rebeaud ME, Mulot M, Guihur A, El Fatouhi D, et al. Diabetes, hypertension, body mass index, smoking and COVID-19-related mortality: a systematic review and meta-analysis of observational studies. BMJ Open. 2021 Oct 25;11(10):e052777. 10.1136/bmjopen-2021-052777 34697120 PMC8557249

[29] Liu L, Ni SY, Yan W, Lu QD, Zhao YM, Xu YY, et al. Mental and neurological disorders and risk of COVID-19 susceptibility, illness severity and mortality: a systematic review, meta-analysis and call for action. EClinicalMedicine. 2021;40:101111. 10.1016/j.eclinm.2021.101111 34514362 PMC8424080

